# Highly *c*-axis orientated superconducting core and large critical current density in Ba_0.6_Na_0.4_Fe_2_As_2_ powder-in-tube tape

**DOI:** 10.1038/s41598-019-49363-y

**Published:** 2019-09-10

**Authors:** S. Imai, S. Itou, S. Ishida, Y. Tsuchiya, A. Iyo, H. Eisaki, K. Matsuzaki, T. Nishio, Y. Yoshida

**Affiliations:** 1 Department of Physics, Tokyo University of Science, 1-3 Kagurazaka, Shinjuku Tokyo, 162-8601 Japan; 2 National Institute of Advanced Industrial Science and Technology (AIST), 1-1-1 Umezono, Tsukuba Ibaraki, 305-8568 Japan

**Keywords:** Superconducting properties and materials, Superconducting properties and materials

## Abstract

Improvement of the critical current density (*J*_c_) of superconducting wires/tapes is one of the key issues in the field of superconductivity applications. Here we report the fabrication of a silver-sheathed Ba_1−*x*_Na_*x*_Fe_2_As_2_ (BaNa-122) superconducting tape by using a powder-in-tube technique and its superconducting properties, in particular transport *J*_c_, as well as the tape-core texture. The optimally-doped BaNa-122 tape with Na concentration *x* = 0.4 exhibits the superconducting critical temperature (*T*_c_) of 33.7 K and high transport *J*_c_ of 4 × 10^4^ A/cm^2^ at 4.2 K in a magnetic field of 4 T. Patterns of x-ray diffraction for the superconducting core show that the degree of *c*-axis orientation is significantly enhanced through the tape fabrication process. The tendency of *c*-axis orientation is advantageous for achieving higher *J*_c_, suggesting the high potential of BaNa-122 for superconducting wire/tape applications.

## Introduction

Since the discovery of the high-transition-temperature (high-*T*_c_) superconductivity in iron-based superconductors (IBSs)^1^, they have attracted much attention from the viewpoint of basic research as well as applications^2–5^. Among various types of IBSs, so-called 122-type materials represented as *AE*_1−*x*_*A*_*x*_Fe_2_As_2_ (*AE*: alkaline earth metal, *A*: alkali metal) have several characteristics suitable for applications^6–8^: the maximum *T*_c_ of 38 K, large upper critical fields (*H*_c2_) exceeding 100 T, and small anisotropy (*γ* = *H*_c2_^*ab*^/*H*_c2_^*c*^ ~ 1–2)^9,10^. In particular, K-doped (*A* = K) materials, i.e. Ba_1−*x*_K_*x*_Fe_2_As_2_ (BaK-122) and Sr_1−*x*_K_*x*_Fe_2_As_2_ (SrK-122), have been generally used for the fabrication of powder-in-tube (PIT) wires/tapes. The latest BaK-122 tapes possess the transport critical current density (transport *J*_c_) of 1.5 × 10^5^ A/cm^2^ at 4.2 K and 10 T^11^, which is over a level in practical use under this condition, while further improvement of *J*_c_ is required.

To date, massive efforts have been devoted to improve the fabrication process of wires/tapes toward higher *J*_c_. It has been established that *J*_c_ values can be significantly increased when a uniaxial pressing is applied to tapes after the flat-rolling process. The application of uniaxial pressure is found to enhance the superconducting-core density as well as the *c*-axis alignment of the grains, which are considered to be correlated with *J*_c_^11–17^. Accordingly, the enhancement of two factors, namely, the core density and the grain alignment, has been mainly focused to achieve higher *J*_c_. Currently, the highest *J*_c_ was achieved for BaK-122 tapes fabricated by a hot-press technique^11^. Here, Huang *et al*.^11^ argued that BaK-122 is more suitable for the PIT tapes compared with SrK-122 because a higher degree of *c*-axis orientation in the core, which was associated with the high *J*_c_, was obtained through the same fabrication process. In addition, the Vickers hardness of the tape core, which is a measure of the core density, was smaller than that of 122 wires (no *c*-axis orientation)^18^. This suggests that the *c*-axis orientation becomes more important to achieve a practical-level *J*_c_ after obtaining a sufficiently high core density.

Meanwhile, we fabricated PIT tapes using Sr_1−*x*_Na_*x*_Fe_2_As_2_ (*AE* = Sr and *A* = Na; SrNa-122) and achieved a high transport *J*_c_ exceeding 1 × 10^4^ A/cm^2^ at 20 K under magnetic fields up to 2.5 T^19^. Interestingly, we found the precipitation of Ag-As alloys in the superconducting core of SrNa-122 tape after the heat treatment process, although Ag was not added to the starting powders. The precipitation of Ag-As alloys has not been reported for any other *AE*K-122 wires/tapes. The Ag-As alloys are likely conductive, hence they possibly improve the grain connectivity and thus contribute to the enhancement of *J*_c_ as discussed in previous studies where extra metals such as Sn, Pb, and Ag were added to the core^17,20^.

These results in previous studies indicate that the choice of *AE* and *A* enhances/suppresses the *c*-axis alignment of the core and/or promotes the chemical reaction with sheath materials (Ag in the present case), and thus affects *J*_c_ of 122-based PIT wires/tapes. In this study, we focus on the combination of *AE* = Ba and *A* = Na, namely, Ba_1−*x*_Na_*x*_Fe_2_As_2_ (BaNa-122)^21^, which is recently used for the PIT wire fabrication^22^, while its potential for the PIT tape fabrication is unexplored. We obtained high *J*_c_ exceeding 4 × 10^4^ A/cm^2^ at 4.2 K under a magnetic field of 4 T. We found that the BaNa-122 tape core has a remarkably high degree of *c*-axis orientation by comparing the orientation factor with various BaK-122 and SrNa-122 tapes. We also found the precipitation of Ag-As alloys in the BaNa-122 tape core similarly to the case of SrNa-122 tape, while the amount of Ag-As precipitation is much smaller compared with SrNa-122. The Vickers hardness of the BaNa-122 tape core (*H*_v_ ~ 90) is relatively small, indicating that the low core density is the main limiting factor of *J*_c_ of the present BaNa-122 tape.

## Results

### X-ray diffraction analysis

**Figure 1:**
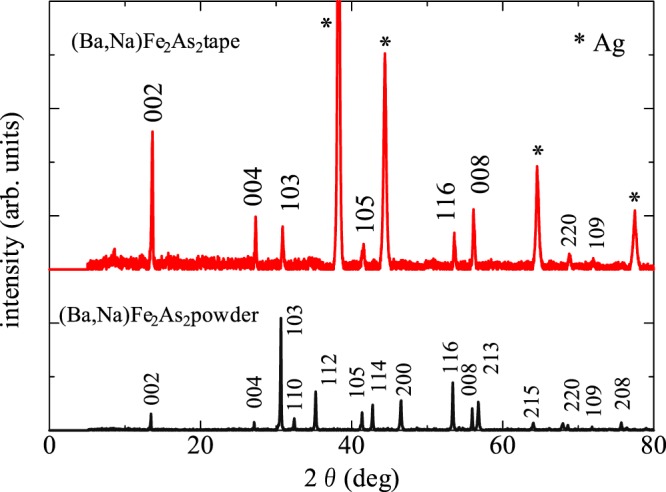
XRD patterns of randomly oriented BaNa-122 polycrystalline powder (black line) and the tape surface (red line). Asterisks indicate diffraction peaks from Ag sheath material.

Figure 1 shows XRD patterns of the BaNa-122 polycrystalline powder (black) and tape (red) samples. All the visible peaks can be indexed based on the 122 structure (ThCr_2_Si_2_ type, I4/mmm). No impurity peaks were observed in the range of resolution. The lattice constants of the powder sample estimated from the *d* values of peaks are *a* = 3.902 Å and *c* = 13.13 Å. These values correspond to *x* = 0.4^21^, indicating that the Na composition of the present powder was successfully controlled. The peak positions of XRD patterns of the superconducting core surface are almost identical to those for polycrystalline powders within the experimental certainty, indicating that the Na concentration was not significantly changed through the tape fabrication process. On the other hand, the intensities of the 00 *l* peaks of tape core are largely enhanced in comparison with the polycrystalline powder. This indicates that the *c*-axis of BaNa-122 grains is well-oriented perpendicularly to the tape surface, that is, along the direction of the uniaxial pressure.

### Magnetic susceptibility

**Figure 2:**
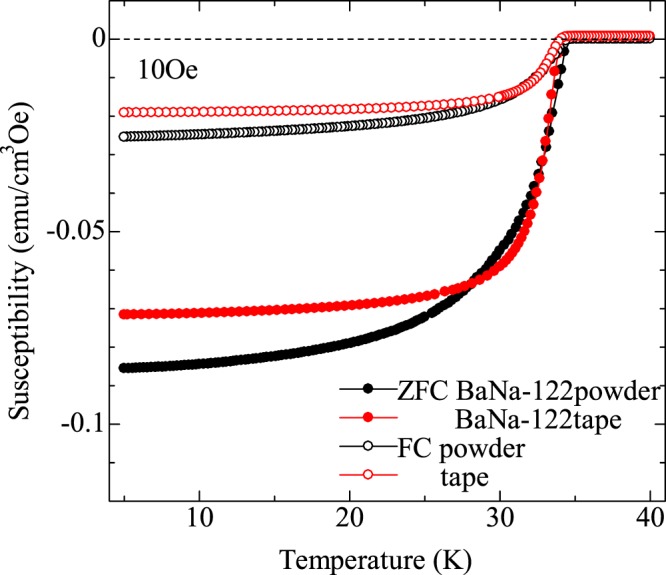
Temperature dependence of magnetic susceptibility of BaNa-122 powder (black) and tape (red) measured under the field of 10 Oe. The filled and open symbols indicate the data taken by the zero-field-cool and field-cool processes, respectively.

Figure 2 shows the temperature (*T*) dependence of magnetic susceptibility for polycrystalline powders (black) and a 3 mm-long tape (red) measured under the field of 10 Oe. The filled and open symbols indicate the data taken by the zero-field-cool and field-cool processes, respectively. The *T*_c_ values were determined as a temperature where the susceptibility dropped by 10% of the superconducting transition in the zero-field-cool branch. The determined *T*_c_ of the polycrystalline powder is 34.2 K, which is in good agreement with the reported *T*_c_ of BaNa-122 with *x* = 0.4^21^, supporting that the Na concentration was successfully controlled. After the fabrication process, *T*_c_ ( = 33.7 K) of the tape is found to decrease by 0.5 K from that of powder, while the width of superconducting transition is preserved.

The degradation in *T*_c_ after wire/tape fabrication process has been also reported in previous works^23^. An explanation for the reduction in *T*_c_ is the loss of Na during the fabrication process, although the Na concentration is seemingly unchanged within the resolution of the XRD and the EDX measurements. On the other hand, the reduction in *T*_c_ is possibly caused by the mechanical stress during the wire/tape fabrication process. It was pointed that *T*_c_ possibly decreases if the local structures such as pnictogen height and As-Fe-As bond angle are changed by the mechanical stress because *T*_c_ of IBSs is sensitive to these local structures.

### Transport *J*_c_

**Figure 3:**
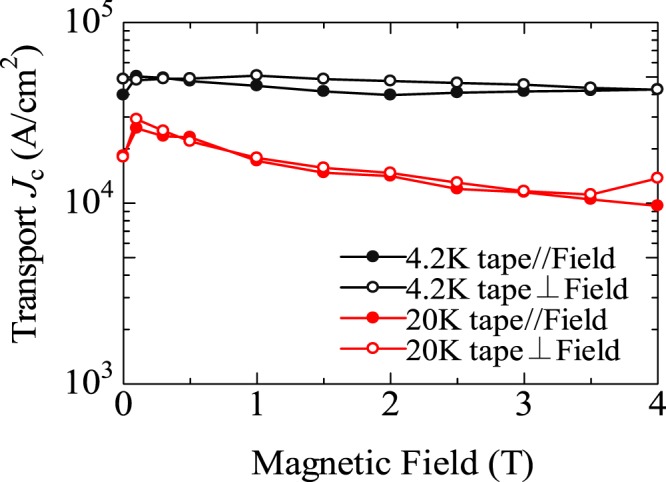
Magnetic field dependence of transport *J*_c_ of a (Ba, Na)Fe_2_As_2_ tape at 4.2 K (black) and 20 K (red) under magnetic fields up to 4 T applied parallel (closed circles) and perpendicular (open circles) to the tape surface.

Figure 3 shows the magnetic field (*H*) dependence of transport *J*_c_. The measurements were performed at 4.2 K (black) and 20 K (red) under magnetic fields up to 4 T applied parallel (filled circles) and perpendicular (open circles) to the tape surface. At *T* = 4.2 K and *H* // tape surface, the BaNa-122 tape shows a large transport *J*_c_ values of 5.0 × 10^4^ A/cm^2^ and 4.3 × 10^4^ A/cm^2^ under self-field and 4 T, respectively. The high *J*_c_ is maintained even at 20 K; *J*_c_ values are 1.5 × 10^4^ A/cm^2^ under a self-field and 1.0 × 10^4^ A/cm^2^ under 4 T. Moreover, *J*_c_ curves exhibit weak *H* dependence with a ratio *J*_c_(0 T)/*J*_c_(4 T) ~ 1.4–1.5. Furthermore, *J*_c_ values under the magnetic fields parallel and perpendicular to the tape surface are almost identical to each other, or *J*_c_ is even slightly higher for *H* perpendicular to the tape surface.

### SEM-EDX analysis

**Figure 4:**
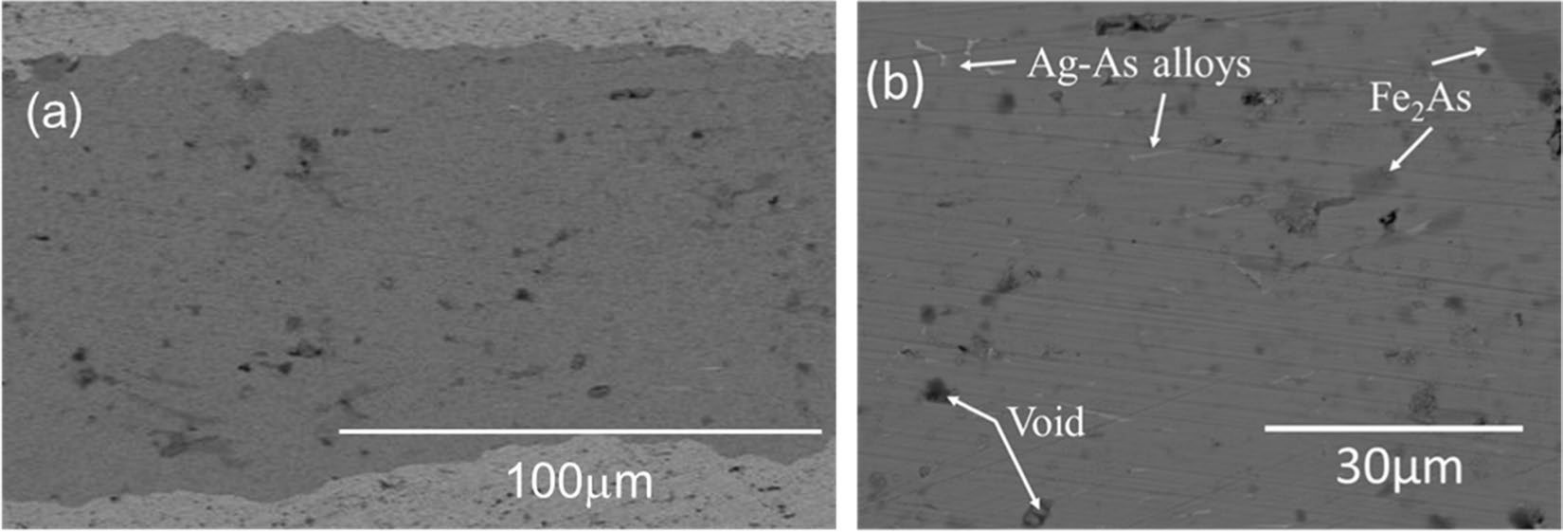
(**a**,**b**) SEM images of the cross section of a BaNa-122 tape taken at different magnifications.

Figure 4(a,b) show SEM images of the cross section of the BaNa-122 tape core obtained at different magnifications. EDX analyses show that the average atomic composition in the core, Ba: Na: Fe: As, is approximately 12: 8.8: 37: 40. This atomic ratio corresponds to (Ba_0.62_Na_0.43_)Fe_1.8_As_2.0_, indicating that the Na concentration is ~0.4, which is close to the nominal composition of the starting powder. The SEM images show the presence of Fe_2_As impurities (dark gray spots), which are not detected by the XRD measurements. The amount of Fe_2_As was estimated to be ~3.7% from the cross-section area of the SEM images. The black regions correspond to voids or cracks with the sizes of a few micrometers. Such voids/cracks have been considered as one of the main limiting factors of *J*_c_. The blight rod-like spots are found to be Ag-As alloys. Note that Ag was not added to the starting powders and a similar phenomenon was reported for the SrNa-122 tape^19^.

## Discussion

**Figure 5:**
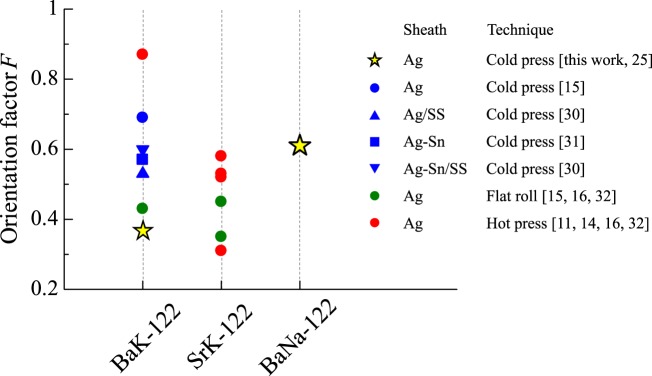
Orientation factor *F* of BaK-122^11,15,25,30,31^, SrK-122^14,16,32^, and BaNa-122. Several *F* values were calculated using the XRD patterns given in the references. The symbols indicate the sheath materials and techniques used for the tape fabrication process.

A high-performance IBS tape was successfully fabricated by using BaNa-122, demonstrating that BaNa-122 is also a good candidate material for superconducting wire/tape applications. In particular, an advantageous characteristic is found, i.e. the superconducting core of the BaNa-122 tape is strongly *c*-axis oriented perpendicularly to the tape surface. Note that the high *c*-axis orientation has been considered as an important factor for improving *J*_c_^14,15^. To quantify the degree of *c*-axis orientation, the orientation factor (*F*) was estimated based on the XRD patterns by using the Lotgering method^24^, i.e. *F* = (*ρ* – *ρ*_0_)/(1 – *ρ*_0_) with *ρ* = Σ*I*(00 *l*)/Σ*I*(*hkl*) and *ρ*_0_ = Σ*I*_0_(00*l*)/Σ*I*_0_(*hkl*), where *I*(*hkl*) (*I*_0_(*hkl*)) is the intensity of the *hkl* peak for tapes (polycrystalline powders). The estimated *F* of the present BaNa-122 tape is 0.61. This value is larger than that of our BaK-122 tape (*F* = 0.37)^25^, suggestive of a better *c*-axis orientation of the BaNa-122 core. For further comparison, in Fig. 5, we plotted the *F* values of various BaK-122 and SrK-122 tapes taken from references. The *F* value of the BaNa-122 tape is found to be considerably high among those of 122 tapes. It should be noted that the high *F* values (>0.5) of previous BaK-122 and SrK-122 tapes were achieved using advanced techniques efficient for obtaining the *c*-axis orientation; (i) application of large uniaxial pressure (~4 GPa, which is 4 times larger than that in this work), (ii) application of hot-press technique, or (iii) use of Ag-Sn alloy sheath or Ag/stainless steel (SS) double sheath (which are harder than the pure Ag sheath used in this work). Thus, the comparison of *F* values between the BaNa-122 tape and various BaK-/SrK-122 tapes demonstrates that the use of BaNa-122 material is promising for obtaining a better *c*-axis orientation of the tape core.

The high *c*-axis orientation found for the BaNa-122 core is possibly associated with the strain in the crystal structure. It is reported that the *c* axis of BaNa-122 shows a non-monotonic *x* dependence violating the Vegard’s law (the *c*-axis parameter increases up to *x* ~ 0.4 while decreases at higher *x*) owing to the large mismatch of ionic radii between Ba^2+^ and Na^+ ^^26^. This suggests that BaNa-122 has a significant strain in the crystal structure compared with other 122 materials, which possibly causes an easy cleavage of the grains during the flat-rolling and uniaxial-pressing processes. Such cleaved grains likely align easily and thus result in the high *c*-axis orientation of the tape core.

In addition, the precipitation of Ag-As alloys was observed in the BaNa-122 tape core similarly to the case of SrNa-122 tape^19^, which has not been reported for K-doped 122 tapes. The Ag-As alloys occupy about 0.6% of the cross-section area for the present BaNa-122 tape, which is smaller than that estimated for the previous SrNa-122 tape (~3%). Meanwhile, the amount of Fe_2_As impurities is also smaller for BaNa-122 (~3.7%) than that for SrNa-122 tape (~10%). For BaK-122 and SrK-122, the Ag-As alloys have not been observed and the amount of Fe_2_As impurities is much smaller. There seems a correlation between the amounts of Ag-As alloys and Fe_2_As impurities, suggesting that the precipitation of Ag-As alloys is the resultant of the chemical reaction between 122 phase and the Ag sheath. The degree of chemical reaction between 122 and Ag sheath is possibly associated with the chemical stability of 122 phase. Note that *AE*Na-122 phase has a solubility limit at some Na concentration because Na-122 (*x* = 1) is a metastable phase^21,27,28^, while *AE*K-122 phase can be obtained for any K concentration. Therefore, *AE*Na-122 is likely more unstable and hence reactive compared with *AE*K-122. In addition, SrNa-122 used for the tape contains a higher Na concentration than that of BaNa-122 because *T*_c_ is optimized at *x* = 0.55 and 0.4 for SrNa-122 and BaNa-122, respectively. Then, SrNa-122 is closer to the solubility limit hence more unstable compared with BaNa-122. Thus, the precipitation of Ag-As alloys is most prominent for the most unstable SrNa-122 core.

Regarding the Ag-As precipitation, two opposing effects on the grain connectivity, which is one of the key factors determining *J*_c_, are considered; (i) the Ag-As alloys fill voids and cracks in the core and thus improve the grain connectivity, and (ii) the accompanying Fe_2_As impurities disturb the grain connectivity. Then, *J*_c_ is possibly increased by balancing the positive and negative effects. For the optimization of the precipitation of Ag-As alloys and Fe_2_As impurities, the fine adjustments of the heat-treatment temperature and time as well as the chemical composition of the BaNa-122 powder should be considered.

Meanwhile, the transport *J*_c_ of the present BaNa-122 tape does not reach the practical-level *J*_c_ (=10^5^ A/cm^2^) in spite of the considerably high *F* value. To understand the limiting factor of *J*_c_, we checked the Vickers hardness (*H*_V_) of the BaNa-122 core, which is often used as a measure of the core density. The obtained *H*_V_ ~ 90 is relatively small compared with the other 122 tapes with higher *J*_c_. Therefore, we concluded that the low core density owing to the presence of crack and voids mainly limits *J*_c_ of the present BaNa-122 tape. Such cracks and voids are expected to be reduced by using the techniques mentioned above, i.e. application of a large uniaxial pressure, hot press, and hard sheath materials. Note that these techniques are also efficient for the enhancement of *c*-axis orientation. Thus, the use of BaNa-122, which provides a better *c*-axis orientation, possibly leads to the further enhancement of *J*_c_ of 122-based PIT tapes. On the other hand, it should be also noted that the cold- and hot-press techniques limit the length of the tape hence they are not suitable for the mass production (in the present study, the length of the tape (~50 mm) is limited by the uniaxial-press process). Then, the application of hot rolling and over-pressure sintering, which have been used for the production of high-*T*_c_ BSCCO tapes, should be interesting in terms of the scale up of the tape length.

Finally, regarding the *H* dependence of transport *J*_c_ of the present BaNa-122 tape, a weak *H* dependence and a small anisotropy against the *H* orientation were observed similarly to BaK-122 and SrK-122 tapes, which are important properties from the application viewpoint. Note that the *J*_c_ anisotropy is smaller than (or inversed from) that expected from the anisotropy of *H*_c2_, namely, the effective mass model. The inverse anisotropy has been also reported for BaK-122^11^ and SrK-122 tapes^29^. To account for this behavior, two scenarios have been proposed; (i) correlated pinning centers, i.e. elongated perpendicular to the tape surface, or (ii) pinning centers larger than the coherence length dominate the pinning properties. To elucidate the origin of the inverse anisotropy of *J*_c_, it is desired to perform the transmission-electron-microscopy measurement, which is a powerful tool to investigate the defect structures determining vortex pinning properties.

## Summary

An iron-based superconducting PIT tape was fabricated using BaNa-122. High *J*_c_ values of 5.0 × 10^4^ A/cm^2^ at 4.2 K under a self- field and 1.0 × 10^4^ A/cm^2^ at 20 K under a magnetic field of 4 T were achieved, which are comparable to those in BaK-122 and SrK-122 tapes. Also, a weak *H* dependence of *J*_c_ and a small anisotropy of *J*_c_ against the *H* orientation were observed similarly to the cases of BaK-122 and SrK-122 tapes, which are important properties for high-field applications. Moreover, we found an advantageous characteristic of BaNa-122 tape; the superconducting core tends to show a high *c*-axis orientation. Meanwhile, a substantial amount of voids and cracks are observed in the core, which are likely to be the main limiting factor of the transport *J*_c_ of present BaNa-122 tape. Then, there still remains room for the further enhancement of *J*_c_ in BaNa-122 tapes by taking advantage of the better *c*-axis orientation.

## Methods

Polycrystalline powders of BaNa-122 were synthesized by a solid-state reaction. Prior to the synthesis, BaAs, NaAs, and Fe_2_As powders were prepared as precursors by mixing elemental Ba, Na, Fe, and As chunks at stoichiometric molar ratio, sealing them into quartz tubes (BaAs and Fe_2_As) or stainless-steel container (NaAs), and heating at 650 °C (BaAs and NaAs) and 750 °C (Fe_2_As). The precursors were mixed in the ratio of BaAs: NaAs: Fe_2_As = 0.6: 0.45: 1. Here, the target Na concentration was *x* = 0.4 so as to yield highest *T*_c_ of 34.2 K. Extra NaAs was added considering the possible loss of Na and As during the synthesis process. The mixed powders were put in an alumina crucible and then sealed in a stainless-steel container, then sintered at 770 °C for 15 hours. The polycrystalline powders were pressed into a pellet by applying a pressure of about 100 MPa. The pellet was packed into a silver tube having an outer diameter of 10 mm and an inner diameter of 4.3 mm. The silver tube was groove-rolled into a square wire with the 1.2 × 1.2 mm^2^ cross-sectional and subsequently flat-rolled into a tape with a thickness of 0.48 mm. The flat rolled tape was uniaxial cold-pressed between stainless steel disks under a pressure of approximately 1.0 GPa. After this press process the thickness of tape was reduced to 0.20 mm. The thin tape was cut into 50 mm long pieces, sealed into a stainless-steel container in a N_2_-filled glove box, and sintered at 875 °C for 3 h.

Measurements of x-ray diffraction (XRD) for polycrystalline powders and superconducting tape cores were performed at room temperature using Cu *K*_α_ radiation (the peaks from Cu *K*_α2_ were removed using Peak search, Rigaku Corporation). Magnetic susceptibility was measured by using a SQUID magnetometer (Quantum design, MPMS). The cross section of the superconducting core was examined with a scanning electron microscope SEM (Hitachi High-Technologies TM3000) and the composition analysis was carried out by an energy dispersive x-ray EDX spectrometer (Oxford Instruments SwiftED 3000). Vickers hardness (*H*_V_) was measured on the polished surface of the tape core using a Micro Vickers hardness testing machine (Matsuzawa MMT-X3). Five different positions were measured with a 25 g load and 10 s duration, and an average value was calculated. Transport critical current (*I*_c_) measurements were performed by using a four-terminal method at 4.2 K and 20 K under magnetic fields up to 4 T.
